# Magnetic Cross-Linked Enzyme Aggregates (mCLEAs) of *Candida antarctica* Lipase: An Efficient and Stable Biocatalyst for Biodiesel Synthesis

**DOI:** 10.1371/journal.pone.0115202

**Published:** 2014-12-31

**Authors:** Álvaro Cruz-Izquierdo, Enrique A. Picó, Carmen López, Juan L. Serra, María J. Llama

**Affiliations:** 1 Department of Biochemistry and Molecular Biology, University of the Basque Country (UPV/EHU), Bilbao, Spain; 2 IKERBASQUE, Basque Foundation for Science, Bilbao, Spain; Oak Ridge National Laboratory, United States of America

## Abstract

Enzyme-catalyzed production of biodiesel is the object of extensive research due to the global shortage of fossil fuels and increased environmental concerns. Herein we report the preparation and main characteristics of a novel biocatalyst consisting of Cross-Linked Enzyme Aggregates (CLEAs) of *Candida antarctica* lipase B (CALB) which are covalently bound to magnetic nanoparticles, and tackle its use for the synthesis of biodiesel from non-edible vegetable and waste frying oils. For this purpose, insolubilized CALB was covalently cross-linked to magnetic nanoparticles of magnetite which the surface was functionalized with –NH_2_ groups. The resulting biocatalyst combines the relevant catalytic properties of CLEAs (as great stability and feasibility for their reutilization) and the magnetic character, and thus the final product (mCLEAs) are superparamagnetic particles of a robust catalyst which is more stable than the free enzyme, easily recoverable from the reaction medium and reusable for new catalytic cycles. We have studied the main properties of this biocatalyst and we have assessed its utility to catalyze transesterification reactions to obtain biodiesel from non-edible vegetable oils including unrefined soybean, jatropha and cameline, as well as waste frying oil. Using 1% mCLEAs (w/w of oil) conversions near 80% were routinely obtained at 30°C after 24 h of reaction, this value rising to 92% after 72 h. Moreover, the magnetic biocatalyst can be easily recovered from the reaction mixture and reused for at least ten consecutive cycles of 24 h without apparent loss of activity. The obtained results suggest that mCLEAs prepared from CALB can become a powerful biocatalyst for application at industrial scale with better performance than those currently available.

## Introduction

Due to the global shortage of fossil fuels, a consequent excessive rise in the price of crude oil and increased environmental concerns, a rapid growth in biodiesel production has being observed [1]. Biodiesel is a clean-burning diesel fuel composed of a mixture of alkyl esters of long-chain fatty acids which is typically produced from nontoxic, renewable biological resources such as vegetable oils, animal fats, or even used cooking oils [2]. Nowadays, the alkali-catalyzed transesterification of triglycerides present in oils or fats is the most common way to produce biodiesel at industrial scale due to its high conversion and kinetics. However, when these raw materials contain an elevated percentage of water or free fatty acids, undesirable reactions drastically reduce the yield and quality of the resulting biodiesel [2]. Thus, enzyme-catalyzed production of biodiesel by lipases is more advantageous over the chemical counterpart since it is chemically selective, reaction is carried out at lower temperature and under milder conditions and downstream processes are more environmentally friendly [3].

Lipases constitute a group of versatile enzymes that catalyze the hydrolysis of lipids in aqueous media, but in organic media they can catalyze synthetic reactions, including interesterifications between triglycerides and alcohols to produce glycerin and alkyl-esters of long chain fatty acids, *i.e.*, biodiesel. However, free lipases are easily inactivated in organic solvents and difficult to recover for reuse [4]. *Candida antarctica* lipase B (CALB) is an extracellular monomeric globular (33.5 kDa) enzyme which is not as efficient as other lipases in hydrolyzing triglycerides, but it is highly stereospecific towards both ester hydrolysis and synthesis. Due to its efficiency and high selectivity this lipase has been immobilized by several methods (especially by covalent attachment to silica gel, celite or activated nanoparticles) and it is being used in a wide range of applications replacing industrial synthetic processes [5].

Many strategies have been recently applied to immobilize CALB. One of the most successful consists of covalent linking of the enzyme to the surface of iron oxide magnetic nanoparticles (MNPs) [6], [7]. The high specific surface of MNPs favors the binding efficiency of the enzyme, and the superparamagnetic behavior of the support permits the easy and selective recovery of the biocatalyst using a magnet and its subsequent reuse in more catalytic cycles [8], [9]. On the other hand, the support-free methodology consisting of the formation of cross-linking enzyme aggregates (CLEAs) of lipase with glutaraldehyde [10], [11] is a simple technique which does not require the use of highly pure enzymes. It has a broad scope and affords robust, recyclable catalysts that exhibit high activity retention, enhanced thermal stability, better tolerance to organic solvents, and enhanced resistance to autoproteolysis due to the rigidification of the tertiary structure of the enzyme [12]–[15]. Due to the large size of the aggregates, CLEAs can be recovered from the reaction medium by centrifugation or filtration, although this process leads to the increase of CLEAs size and formation of clusters (clumping) resulting in internal mass-transfer limitations [16], [17].

In recent years, lipases immobilized on MNPs or in CLEAs have been successfully used to obtain biodiesel. Xie and Ma [18], [19] reported 90% conversion to biodiesel using covalently immobilized *Thermomyces lanuginosus* lipase on amino-functionalized MNPs. Also *Burkholderia cepacia* lipase immobilized to MNPs of Fe_3_O_4_ yielded complete conversion to biodiesel [20]. In addition biodiesel was obtained from olive oil using CLEAs of crude preparations from *Photobacterium lipolyticum* lipase M37 [21]. Lai et al. [22] also reported 85.7% conversion to biodiesel from microalgal oil using an ionic liquid (1-butyl-3-methylimidazolium hexafluorophosphate) as a solvent and CLEAs obtained from crude preparations of *Penicillium expansum*.

Recently, our group has developed a method for the preparation of magnetic cross-linked enzyme aggregates (mCLEAs) of CALB. This method consists of the cross-linking of insolubilized lipase to aminated MNPs by glutaraldehyde [23]. The resulting mCLEAs fulfill the main benefits of magnetic biocatalysts and CLEAs, as they show improved thermal and storage stabilities, and can be reused after their recovery from the reaction mixture with a magnet. Also mCLEAs of α-amylase from *Bacillus* sp. [16] and lipase from *Aspergillus niger* [24] were successfully prepared and used to hydrolyze starch and to obtain glycerol carbonate, respectively.

In this paper, the main magnetic properties of mCLEAs were assessed as well as the utility of this robust biocatalyst to obtain biodiesel from several non-edible vegetable oils. Finally, the enhanced stability of our mCLEAs of CALB was ascertained by reusing the magnetic enzyme to catalyze the production of biodiesel in consecutive 24 h batch reactions. A similar procedure to that detailed here to prepare mCLEAs of CALB can be easily expanded to prepare mCLEAs of other enzymes of interest.

## Materials and Methods

### Materials

CALB L, the lipase B of *Candida antarctica* (CALB, EC 3.1.1.3, 19.1 *U*/mg protein; 7.50 mg protein/ml) was kindly provided by Novozymes (Bagsvaerd, Denmark). HyperLadder I markers were purchased from Bioline (Randolph, MA, USA). Tween 20 and Tween 80 were obtained from Panreac (Barcelona, Spain). 3-Aminopropyltriethoxysilane (APTS), NaBH_4_, FeCl_2_, FeCl_3_, dimethylsulfoxide (DMSO), sodium dodecyl sulphate (SDS), *p*-nitrophenyl acetate (*p*NPA), bovine serum albumin, and Triton X-100 were purchased form Sigma-Aldrich (St. Luis, MO, USA). Coomassie Blue was obtained from GE Healthcare (Uppsala, Sweden). All other chemicals were supplied by Merck (Darmstadt, Germany). Non-edible oils (unrefined soybean, jatropha and cameline) were obtained from Bunge Ibérica, S.A. (Zierbena, Spain), Jatropha Hispania, S.L. (Toledo, Spain) and Camelina Company (Madrid, Spain), respectively. Olive oil used as a control was purchased from Carbonell (Madrid, Spain) and waste frying oil was obtained from local restaurants.

### Synthesis and functionalization of magnetic nanoparticles (MNPs)

Magnetic nanoparticles of magnetite (Fe_3_O_4_) were synthesized by coprecipitation of iron salts in alkaline medium [25]. An aqueous solution, 50 ml, containing 0.36 M FeCl_2_ and 0.72 M FeCl_3_ was pumped at 5.0 ml/min (LKB Pump P-1, Pharmacia, Uppsala, Sweden) into 450 ml of 1 M NH_4_OH solution under continuous mechanic stirring at room temperature (S1 Video). The obtained black precipitate was separated from the liquid phase using a magnetic field, and then magnetically washed thrice with ultrapure water (MilliQ, Millipore Co., Bedford, MA, USA) and twice with phosphate buffered saline (PBS, 100 mM sodium phosphate, 150 mM NaCl, pH 7.4). All solutions used for the synthesis of MNPs were prepared using MilliQ water. Moreover, a N_2_ gas stream was continuously bubbled through all solutions during the process. The resulting magnetic nanoparticles contained –OH groups on their surface [26], [27]. In order to functionalize them with –NH_2_ groups, MNPs (300 mg, dry weight) were incubated with 30 ml of 2% (v/v) APTS at 70°C with orbital shaking (200 rpm). After 24 h, they were washed thrice with PBS and maintained in the same buffer at 4°C until use.

### Characterization of MNPs

Phase identification was done by X-ray diffraction studies (XRD, Philips diffractometer PW1710, Almelo, The Netherlands). The crystallite size was calculated from the X-ray line broadening using Scherrer's and Warren's equation:
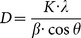
where *K* takes the value of 0.89 considering spherical particles; *λ* is the wavelength of *X* radiation used (0.54 nm); *θ* is the Bragg angle; *β* is the net diffraction peak broadening in radians being determined from:



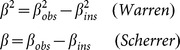
where *β_obs_* is the observed broadening (experimental) and *β_ins_* the instrumental broadening.

The particle size and morphology were determined by transmission electron microscopy (TEM, JEOL 1010, Peabody, MA, USA). The density of –NH_2_ groups on the nanoparticles surface was determined by a colorimetric assay using *p*-nitrobenzaldehyde [28]. The magnetic characteristics were measured using different devices: for the calculation of magnetic saturation (*M_s_*) 2 K hysteresis loops were performed using a vibrating sample magnetometer (VSM) in a superconducting magnet (14 T) cooled by a closed circuit of He (CFMS, Cryogenic Ltd., London, UK). To calculate coercivity (*H_c_*) and remanence (*M_r_*) an electromagnet was used at room temperature and moderate fields (0.9 T). The average size of magnetic particles was calculated from the magnetization data according to Langevin's equations adjusted for non-interacting superparamagnetic model. MNPs dry weight and concentration were analyzed using a vacuum concentrator (Savant SpeedVac concentrator, Thermo Scientific, Waltham, MA, USA).

### Preparation of magnetic CLEAs from CALB

In order to obtain magnetic CLEAs, 10 mg (dry weight) of MNP–NH_2_ were incubated with different concentrations of CALB (from 0.025 to 1.2 mg protein/mg MNP–NH_2_) in the presence of precipitant agents and detergents in a final volume of 15 ml. The precipitant agents used were 80% (v/v) ethanol, 80% (v/v) 2-propanol, 80% (v/v) acetone in water at 4°C, or ammonium sulphate at concentration in the range of 12.5 to 125 mmol/mg protein at 25°C. The detergent concentrations used were 0–5 mM Triton X-100, 0–0.1 mM Tween 20, 0–0.1 mM Tween 80 or 0–25 mM SDS. In all cases, the samples were mixed continuously at 30 rpm using an Intelli-Mixer RM-2, Elmi Ltd. (Riga, Latvia). After 5 min, a 2.5 M glutaraldehyde solution was added (final concentration from 0 to 500 mM) and the suspension was maintained in agitation for 1 to 24 h. After this time, the mCLEAs were removed from the liquid phase using magnetic field, and mCLEAs were washed thrice with PBS. Both the Schiff's bases and the unreacted aldehyde groups were reduced for 2 h at room temperature with rotational mixing with NaBH_4_ (0.75 mg/mg MNP–NH_2_) dissolved in 100 mM carbonate/bicarbonate buffer, pH 10.0. In order to minimize non-specific interactions mCLEAs were then magnetically washed for 10 min firstly with 2 M NaCl in PBS and then with 1% (v/v) Triton X-100 in PBS as reported by Mateo et al. (2000) [29]. After each step, the excess of reagents and possible unretained enzyme were removed by washing thrice with PBS for 10 min. At the end of the immobilization process, the final concentration was 2 mg mCLEAs/ml. The magnetic catalyst was stored at 4°C in PBS for future use.

Magnetic biocatalysts at different formation steps were incubated at 100°C for 5 min, and then, the liquid phase was collected and analyzed by sodium dodecyl sulphate polyacrylamide gel electrophoresis (SDS-PAGE) [30] using homogeneous 8% acrylamide gels revealed with silver (Bio-Rad, Hercules, PA, USA). The amount of immobilized lipase was calculated by measuring the protein remaining in solution at 595 nm (Bio-Rad protein Assay kit, Hercules, PA, USA).

### Enzyme assays

#### Esterase activity with pNPA

The activity of soluble and immobilized CALB was assayed with *p-*nitrophenyl acetate (*p*NPA) as a substrate according to the protocol reported by Gao et al. [31] with minor modifications. Specifically, soluble or immobilized lipase (1–2 µg protein) was added to a reaction mixture which contained 10 µl of *p*NPA (100 mM in DMSO) in 980 µl of PBS. The reaction mixture was maintained for 15 min at room temperature with rotational mixing at 30 rpm (Intelli-Mixer RM-2, Elmi Ltd., Riga, Latvia). Samples were withdrawn every 5 min (in the case of immobilized lipase the magnetic biocatalysts were separated from the liquid phase using a magnet) and the appearance of *p*-nitrophenol (*p*NP) was measured spectrophotometrically (Beckman Coulter DU 800, Brea, CA, USA) at 405 nm (ε*_p_*_NP_ = 9.43 mM^−1^·cm^−1^). One unit (*U*) of esterase activity was defined as the amount of enzyme that catalyzes the appearance of 1 µmol *p*NP per min under the assay conditions.

#### Transesterification of olive oil

Olive oil (0.2 g) and 2-propanol (1∶6 molar ratio oil∶alcohol) were incubated with 1% (w/w of oil) of magnetic catalyst. The reaction was maintained at 40°C and 30 rpm with rotational mixing. The initial reaction rate for biodiesel production was assessed by withdrawing aliquots (5 µl) of the liquid phase at defined intervals which were analyzed by high-performance liquid chromatography (HPLC) as indicated below. Transesterification activity was defined as mg of oil transformed to biodiesel (fatty acid propyl esters, FAPEs) per mg of MNP-NH_2_ and time (h) considering the initial reaction rate of transesterification.

#### Synthesis of biodiesel

FAPEs were produced from vegetable oil (0.2 g) using 2-propanol as alkyl donor (1∶6 molar ratio oil∶alcohol) and 1% (w/w of oil) of magnetic catalyst in a solvent-free reaction. The reaction was followed by withdrawing aliquots (10 µl) of the liquid phase. Semi-quantitative analysis of samples was performed by thin layer chromatography (TLC) and the quantitative analysis using control olive oil was performed by HPLC as described below.

### Analytical methods

#### HPLC analysis

Five µl of samples withdrawn from the reaction mixture were diluted in 250 µl of *n*-hexane and analyzed by HPLC (Waters Corporation, Milford, MA, USA) using a diode array detector according to Holčapek et al. [32]. The C_18_ column (5 µm, 4.6×250 mm, Tracer Lichrosorb RP18) was eluted with 100% pure methanol (phase A) and a mixture of propanol∶*n*-hexane (5∶4 v/v) (phase B) at a flow rate of 0.5 ml/min using a gradient from 0 to 50% phase B in 25 min. Peaks were detected at 205 nm.

#### Thin layer chromatography (TLC) analysis

A semi-quantitative analysis of non-edible or waste frying olive oil was also assessed using TLC. Silica gel 60 coated plates (Merck, Darmstadt, Germany) were activated for 30 min at 100°C and 0.5 µl samples were applied. The ternary mixture *n*-hexane∶ethyl acetate∶acetic acid (90∶10∶1 v/v/v) was used as the eluting phase [33]. After chromatography development (about 40 min), plates were air dried at room temperature, and then immersed for 1 min with gentle orbital shaking in a solution of 0.02% (w/v) Coomassie Blue R-350 [34], prepared in acetic acid∶methanol∶H_2_O (1∶3∶6 v/v/v) as indicated by the manufacturer. Finally, the plates were air dried at room temperature. Spots corresponding to substrates and products of the transesterification reaction were identified by using appropriate reference external standards (triolein, 1,3-diolein, 1-oleyl-rac-glycerol, oleic acid and propyl oleate) run in parallel.

## Results and Discussion

### Magnetic nanoparticles characterization

**Figure 1:**
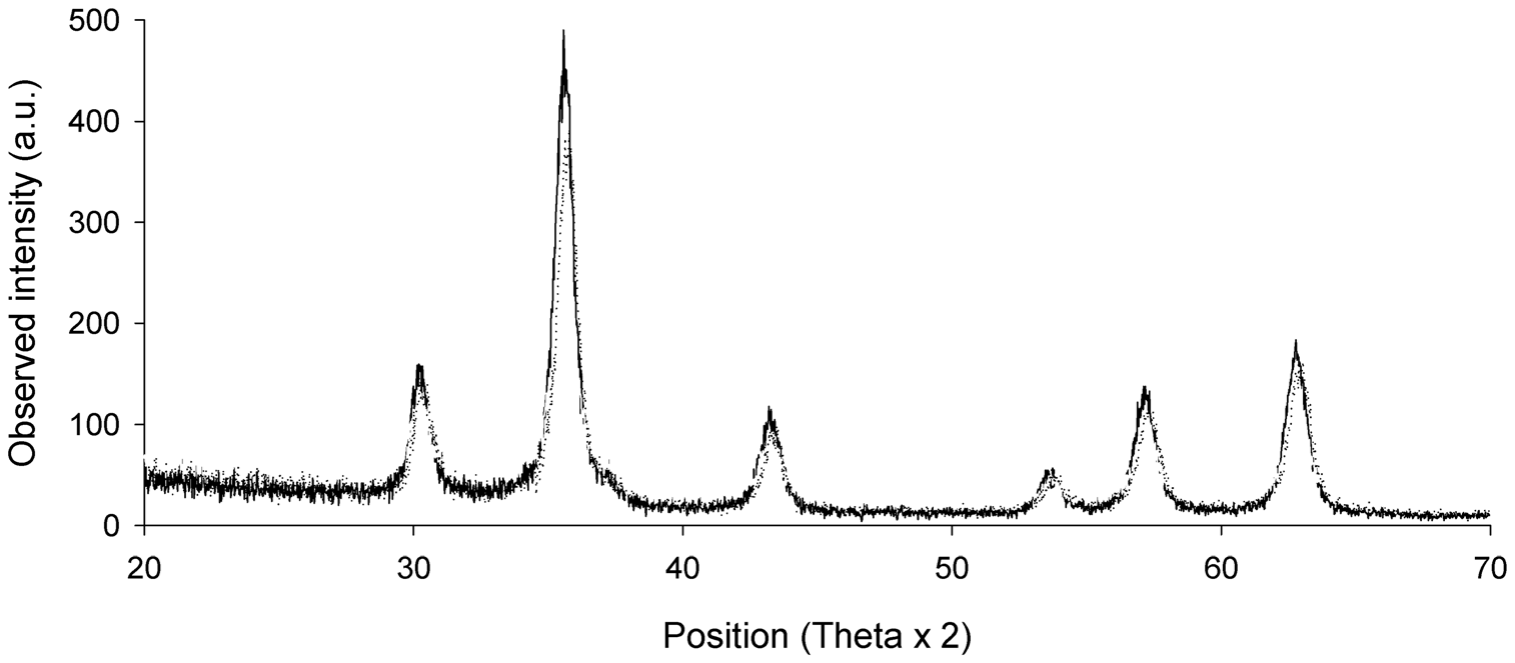
XRD spectrum of magnetic nanoparticles. MNPs were coated with –OH (solid line), and –NH_2_ (dotted line) groups.

**Figure 2:**
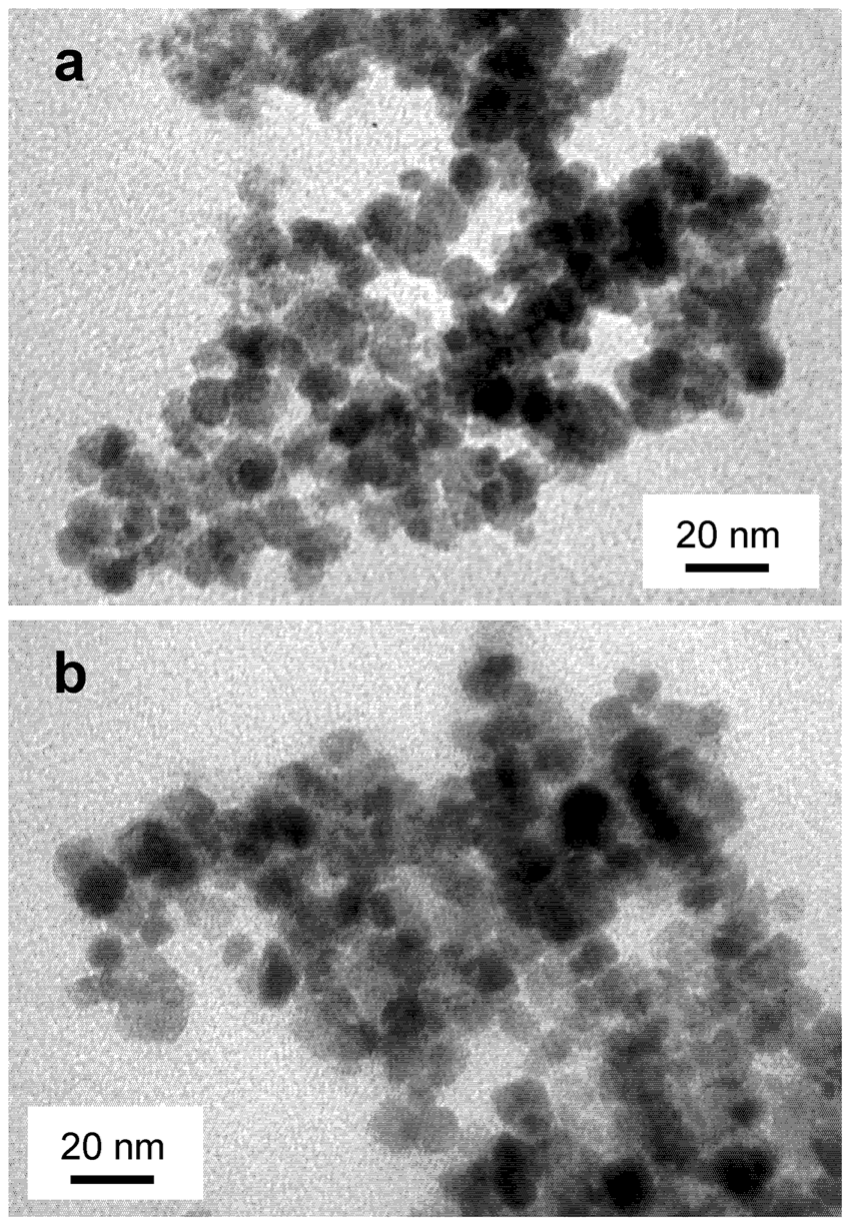
TEM images of magnetic nanoparticles. MNPs were coated with (a) –OH, and (b) –NH_2_ groups. Samples were dispersed in ethanol using an ultrasound bath and then placed onto a Formvar-covered copper grid and evaporated in air at room temperature. Bars represent 20 nm in both pictures.

MNPs size was analyzed by XRD and TEM. X-ray diffraction patterns showed that magnetite constitutes the only crystalline phase present both in MNP–OH and MNP–NH_2_ (Fig. 1), with a crystallite size of 11.2 nm and 11.1 nm, respectively, according to Scherrer's equation, and 9.5 nm and 9.4 nm, respectively, according to Warren's equation. These results indicated that functionalization of MNPs with –NH_2_ groups did not change the phase, nor the crystallite size of the magnetic support. The shape and size of the MNPs was confirmed by TEM (Fig. 2), which showed that both types of MNPs are spherical particles with a mean diameter around 10 nm, indicating that coating their surfaces with –NH_2_ groups did not result in observable differences in their size, shape or aggregation state.

The surface areas were theoretically calculated from the particle size distribution provided by TEM data (Table 1). The results were similar to those reported by other authors, as Harris et al. [35] who determined a surface area of 110 m^2^/g for 8.7 nm nanoparticles, or Sahoo et al. [36] who calculated 93 m^2^/g for particles with mean diameter of 10–12 nm.

**Table 1:** Main characteristics of MNPs coated with –OH and –NH_2_ groups.

	MNP–OH	MNP–NH_2_
Diameter (nm)	10.4±0.9	10.3±0.9
Surface area (m^2^/g)	112.7±9.3	113.8±9.4
–NH_2_ group density (µmol/g)	0.24±0.01	42.96±0.02
*M_s_* (emu/g)	86.0	82.5
*H_c_* (Oe)	0.3	0.1
*M_r_* (emu/g)	3.4	1.0

The density of –NH_2_ groups on the surface of particles was determined using naked MNPs as a control (Table 1). The calculated density of –NH_2_ groups on functionalized nanoparticles (43 µmol/g) was very similar to that reported by del Campo et al. [28] who found approximately 45 µmol/g of MNPs. These authors also calculated the density of –NH_2_ groups by elemental analysis obtaining higher values. This result may suggest that some –NH_2_ groups do not react with 4-nitrobenzaldehyde, and therefore all the amine groups are not available for the interaction with the enzyme. In this case, the calculated density would represent the functionally available groups for the enzyme immobilization.

**Figure 3:**
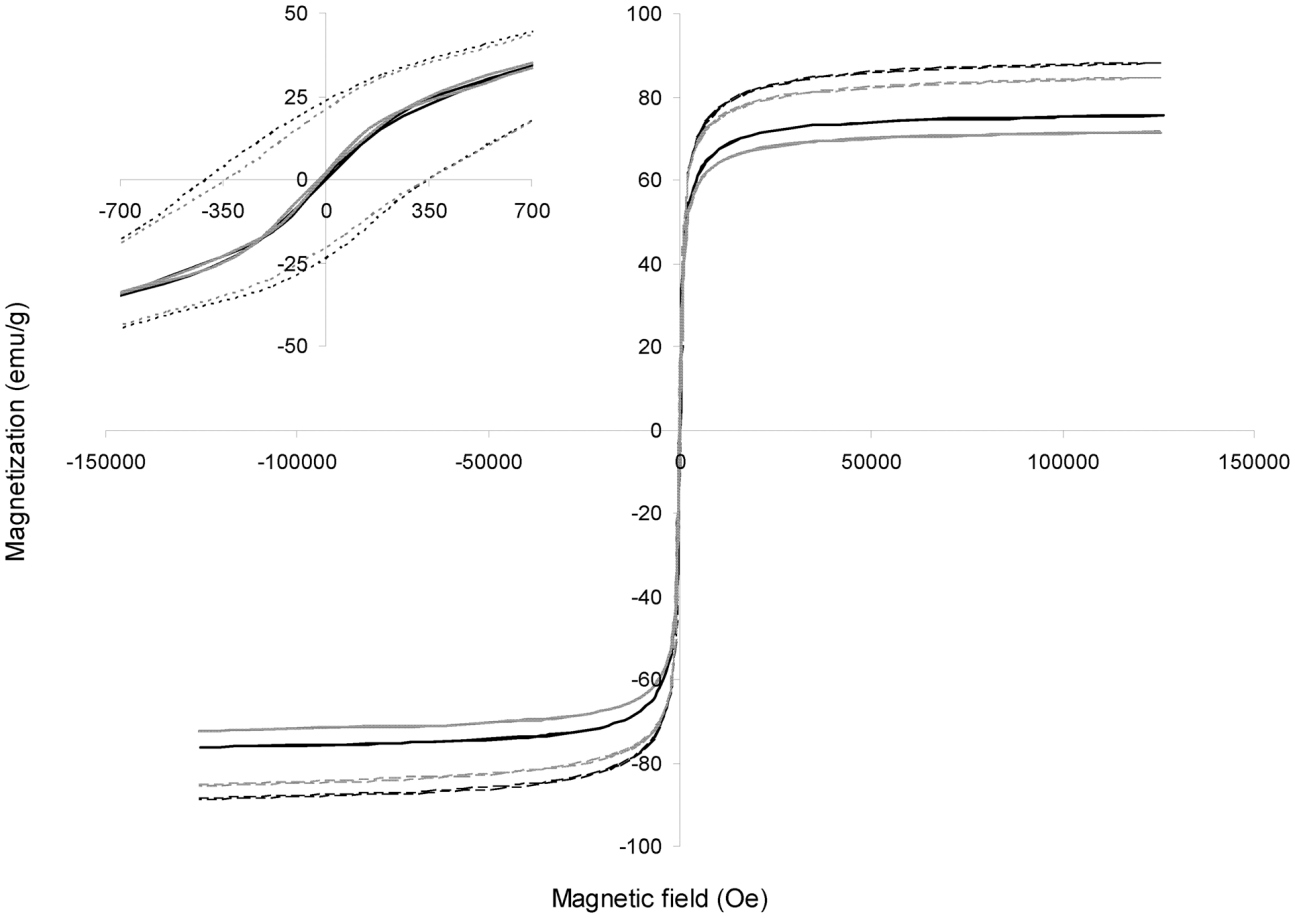
Magnetization analysis of magnetic nanoparticles at different temperatures. Magnetization analysis of MNP–OH (black lines) and MNP–NH_2_ (grey lines) were performed at 300 K (solid lines) and 2 K (dotted lines). Inlet shows a detail of magnetization for values of magnetic field near to 0 Oe.

Magnetic behavior of MNPs was analyzed at different magnetic fields and two temperatures: one below the blocking temperature of magnetite nanoparticles (i.e., the temperature below which magnetite nanoparticles show ferromagnetic characteristics, and corresponds to 75 K, [37]) and the other one at close to room temperature (Fig. 3). Both types of nanoparticles (MNP–OH and MNP–NH_2_) showed hysteresis behavior below the blocking temperature, although this characteristic disappeared at 300 K. At this temperature, magnetic remanence (*M_r_*) and coercivity (*H_c_*) values fell to near zero, which is typical of superparamagnetic nanoparticles. The saturation magnetization (*M_s_*) was determined to be 86.0 and 82.5 emu/g for naked and NH_2_–functionalized nanoparticles, respectively, which is lower than that reported for bulk magnetite (89 emu/g, [38]). This decrease in *M_s_* was also observed by other authors [26], [39] and could be related to the difference between macroscopic and nanoscopic magnetite [25], [27], [35], [39]. On the other hand, the binding of APTS to the nanoparticles caused a slight decrease of 4% in the *M_s_* of the magnetic support, which has been previously observed when organic molecules were immobilized on MNPs [40], [41]. This fact could be explained because the binding of APTS on the nanoparticle surface might quench the magnetic moment [42], [43]. Fitting magnetic result to the Langevin's function, a magnetic core average diameter of 7.8 nm, with 3–4 nm dispersion, was calculated. This value is consistent with the size determined from XRD data as aforementioned.

### Preparation of mCLEAs

Synthesis of mCLEAs involves a sequence of steps starting with enzyme insolubilization followed by cross-linking the resulting protein aggregates with themselves and the surfaces of NH_2_-functionalized magnetic nanoparticles. Optimization aspects concerning the selection of precipitation conditions, cross-linking agent, time or enzyme concentration were considered.

#### Effect of precipitating agent

Commonly used lipase precipitating agents were selected to insolubilize CALB, i.e., ethanol, 2-propanol, acetone and ammonium sulphate (125 mmol/mg protein). After enzyme precipitation, mCLEAs were prepared at room temperature by cross-linking (for 24 h) 0.2 mg CALB/mg MNP–NH_2_ in the presence of 200 mM glutaraldehyde. A control using soluble enzyme was carried out in parallel, with initial protein concentration of 0.133 mg/ml and esterase activity of 2.532 *U*/ml. The end of the immobilization process was verified by analyzing the protein concentration in the liquid phase of the immobilization medium, and the esterase activity in both the liquid and formed mCLEAs. The selected precipitating agents allowed the formation of mCLEAs with 100% protein and esterase activity retention, with the exception of ethanol, which caused a drop in both the retained protein and esterase activity to 46.3% and 46.8%, respectively. In addition to ethanol, also 2-propanol and acetone strongly affected the esterase activity of the biocatalyst, resulting in values of 0.826, 0.655 and 0.383 *U*/mg MNP–NH_2_. However, the mCLEAs obtained with ammonium sulphate showed an activity of 1.823 *U*/mg MNP–NH_2_. When comparing these values with the theoretical immobilized activity (3.8 *U*/mg MNP–NH_2_), immobilization efficiencies of 21.7, 17.2, 10.1 and 48.0% were obtained when using ethanol, 2-propanol, acetone and ammonium sulphate, respectively. These results are in agreement with those reported by other authors [10], [11], [44], who proposed that ammonium sulphate was the best option because it usually preserves the enzymatic activity of the biocatalyst. Therefore, ammonium sulphate was selected in this work as the precipitating agent to prepare mCLEAs of CALB.

**Figure 4:**
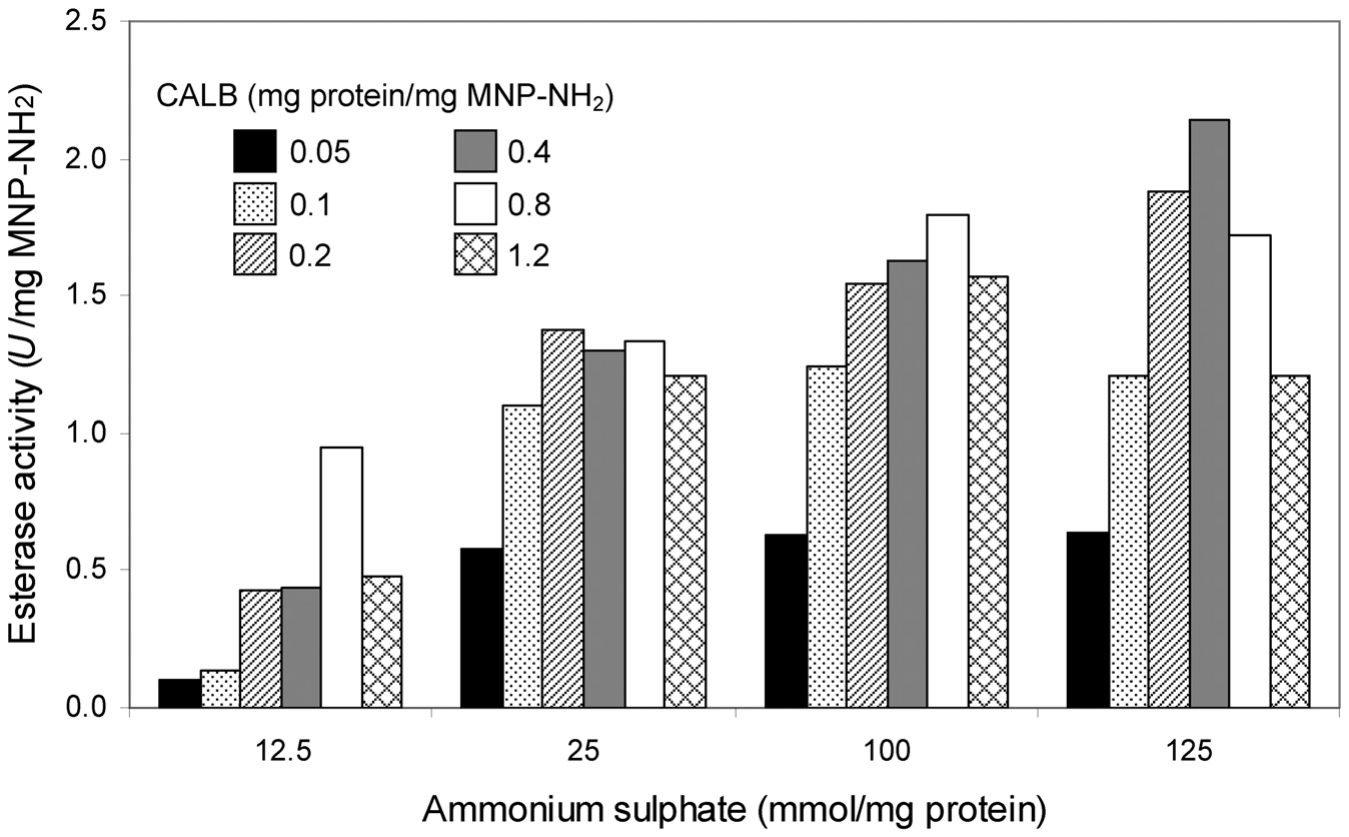
Combined effect of CALB and insolubilizing agent concentrations on the esterase activity of mCLEAs. Different concentrations of lipase were cross-linked for 5 h with 200 mM glutaraldehyde in the presence of the indicated concentrations of ammonium sulphate.

The insolubilizing effect of ammonium sulphate concentration (12.5–125 mmol/mg protein) was studied at room temperature by measuring the esterase activity retained by the resulting mCLEAs prepared using 50 µg to 1.2 mg lipase/mg MNP–NH_2_ and cross-linked for 5 h with 200 mM glutaraldehyde. At low concentration of lipase a good relationship between the immobilized esterase activity and ammonium sulphate was observed (Fig. 4). However at the highest concentration of salt (125 mmol/mg protein), lower activity recovery was measured at lipase concentrations higher than 0.4 mg protein/mg MNP–NH_2_. This effect is probably due to excessive cross-linking and/or size of aggregates which would lead to irreversible enzyme denaturation and/or the appearance of diffusional problems.

It should be noted that the theoretical immobilized activity increases with the increase of enzyme concentration in the preparation. Maximum esterase activities of 0.95, 1.91, 3.82, 7.67, 15.27 and 22.91 *U*/mg MNP–NH_2_ correspond to protein concentrations of 0.05, 0.1, 0.2, 0.4, 0.8 and 1.2 mg protein/mg MNP–NH_2_, respectively. Maximum immobilization efficiencies of around 60% were obtained when 0.1 mg protein/mg MNP–NH_2_ were used. Further increase in enzyme concentrations was not reflected in an increase of recovery activity. This is a usual behavior of immobilized enzymes which is related to diffusional limitations occurring when diffusional rate of substrates and products is lower than reaction rate, thus decreasing the observed rate of the reaction.

Conclusively, 0.1 mg protein/mg MNP–NH_2_ and 100 mmol of ammonium sulphate/mg protein were selected in order to prepare mCLEAs of CALB.

#### Effect of detergents

It is well known that the presence of a detergent at an appropriate concentration can often hyperactivate the enzyme, especially when using lipases [45]–[47]. Furthermore, detergents may prevent the enzyme immobilization through its active center and promote the enzyme orientation in the support so enhancing its activity and/or stability.

The esterase activity of soluble CALB was measured in the presence of Triton X-100, Tween 20, Tween 80 and SDS, at different concentrations below and above their critical micelle concentration (cmc) which were 0.6, 0.06, 0.012 and 8 mM (0.388, 0.074, 0.016 and 2.307 kg/m^3^), respectively. The activity of soluble CALB increased in the presence of Triton X-100 at concentrations above its cmc (S1A Fig.). However, the presence of Triton X-100 at such concentrations during the preparation of mCLEAs had a negative effect in oil transesterification (S1b Fig.). Fernández-Lorente et al. [45] also immobilized CALB in the presence of Triton X-100 on an aminated support, with a final detergent concentration of 1% (v/v) (*i.e.*, 17 mM, 11 kg/m^3^), observing an increase in hydrolytic activity, but this was not tested in organic media [45], [46]. The detergent could enhance enzymatic activity in aqueous medium reactions, but it could lose the hyperactivation capability in hydrophobic media, due to the formation of inverse micelles.

Both Tween 20 and Tween 80 had no effect on CALB esterase activity nor on mCLEAs transesterification activity. The presence of SDS decreased the activity of poorly concentrated mCLEAs, whereas apparently caused a strong hyperactivation of highly concentrated samples. Such hyperactivation is similar to that described by López-Serrano et al. [48] and Gupta et al. [44], who prepared CLEAs in the presence of SDS and observed that the activity increased 2–3 fold. Actually, the transesterification activity of the biocatalyst prepared with high concentration of protein in absence of SDS should be 4-fold higher than the one with lower concentration. The presence of SDS when preparing mCLEAs with highly concentrated aggregates could protect the hydrophobic areas close to active centers, resulting in biocatalysts with lower steric hindrance and thus, higher values of specific activity.

The use of detergents during the formation of mCLEAs would only be recommended for the immobilization of high protein concentrations. However, detergents were not used for further experiments in this work as concentrations of protein above 0.1 mg/mg MNP–NH_2_ seem to result in low immobilized enzyme retentions.

#### Effect of cross-linker concentration

The cross-linking effect of glutaraldehyde was investigated by assessing the esterase activity of mCLEAs prepared by incubating for 5 h the insolubilized lipase (0.1 mg CALB/mg MNP–NH_2_ with 100 mmol ammonium sulphate/mg protein) in the presence of glutaraldehyde at concentrations from 0 to 500 mM (Table 2). The immobilized esterase activity increased linearly when glutaraldehyde concentration was varied from 0 to 200 mM, the maximum value (1.24 *U*/mg MNP–NH_2_) representing 65% of offered activity (1.91 *U*/mg MNP–NH_2_). Above that concentration, the immobilized activity remained unchanged. At higher glutaraldehyde concentration, the cross-linking of MNP–NH_2_ could be favored, reducing the available surface area for the immobilization of aggregates. On the other hand, the immobilization time of 5 h could be insufficient for the formation of covalent bonds, and a more prolonged incubation at these conditions should be used in order to increase the activity retention.

**Table 2:** Effect of cross-linker concentration on the preparation of mCLEAs of CALB.

Glutaraldehyde (mM)	Recovered activity (*U*/mg MNP–NH_2_)	Apparent retained activity (%)
0	0.021	1.1
5	0.019	1.0
10	0.031	1.6
25	0.079	4.1
75	0.391	20.5
125	0.893	46.8
200	1.238	64.8
250	1.188	62.2
300	1.180	61.8
350	1.285	67.3
400	1.315	68.8
500	1.142	59.8

#### Effect of protein concentration and immobilization time

At first approach, mCLEAs of CALB (0.1 mg protein/mg MNP–NH_2_, 100 mmol sulphate/mg protein, 200 mM glutaraldehyde, no detergent) were prepared by varying the time (0, 0.5, 1, 2, 3, 4, 5, 6 and 24 h) used to cross-link the enzyme aggregates onto the surface of MNP–NH_2_. Addition of the cross-linker agent resulted in an immediate activity retention of 42%. This value rose from 65% to almost 100% when the contact time was enlarged from 5 h to 24 h (Table 3). This increase indicates that the immobilization process requires large periods of time to completely establish covalent linkages between enzyme and support. With this in mind, the observed apparent low value of retained activity when high loads of protein were immobilized for 5 h (Table 3) could be due to low retention of protein during this insufficient period of time. When an immobilization time of 24 h was used, the complete protein immobilization was confirmed by analysis of unbound protein at the different steps of the process (S2 Fig.). Even so, high protein loads resulted in low apparent retained activities. Data suggest that 0.1 mg protein/mg MNP–NH_2_ is the maximum concentration that ensures the absence of steric hindrance observed in esterase activity measurement.

**Table 3:** Effect of protein concentration and immobilization time on the preparation of mCLEAs of CALB.

Immobilization time (h)	Offered protein (mg/mg MNP–NH_2_)	Offered activity (*U*/mg MNP–NH_2_)	Recovered activity (*U*/mg MNP–NH_2_)	Apparent retained activity (%)
5	0.05	0.95	0.63	66.0
	0.1	1.91	1.24	65.0
	0.2	3.82	1.55	40.5
	0.4	7.67	1.63	21.3
	0.8	15.27	1.79	11.7
	1.2	22.91	1.57	6.9
24	0.05	0.95	0.93	97.4
	0.1	1.91	1.90	99.5
	0.2	3.82	1.82	47.8
	0.4	7.67	2.08	27.2

Lipases have been immobilized in a great variety of supports in several works [10], [49], obtaining immobilization efficiencies below 80%. Xie and Ma [18], [19] immobilized lipase on amino-functionalized magnetic nanoparticles and studied the optimization of the protein concentration to obtain maximum immobilization efficiencies. As a result, they obtained 70% efficiency at concentrations in the range of 0.025 to 0.05 mg protein/mg MNP–NH_2_. Our improved immobilization method allowed the load of a higher concentration of protein per mg of support with total activity retention, which leads to enzyme and support savings, and consequently economical benefits in production processes.

#### Morphology of mCLEAs

**Figure 5:**
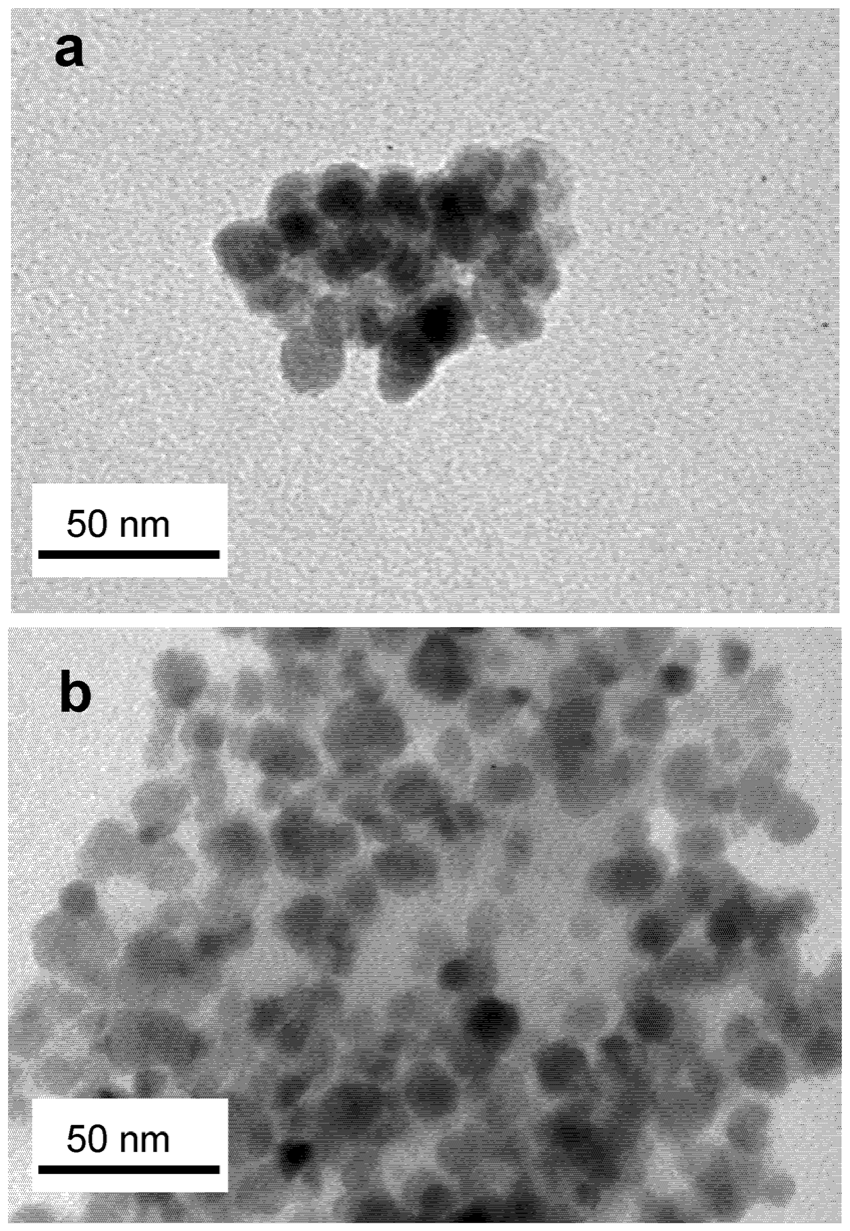
TEM images of mCLEAs. Samples were negatively stained with phosphotungstic acid and dispersed in ethanol as in Fig. 2. (a) and (b) represent different images of the same sample. Bars represent 50 nm in both pictures.

The size and shape of mCLEAs of CALB were examined by TEM after staining the samples with 1% (w/v) phosphotungstic acid. Discrete arrangements of about 100 nm×75 nm (Fig. 5a) were routinely detected although larger aggregates could be also observed (Fig. 5b).

### Biodiesel production by mCLEAs

The ability of the magnetic CLEAs to catalyze the production of biodiesel from vegetable oils in a solvent-free system was tested. The transesterification reaction was performed using 200 mg olive oil and 2-propanol in a molar ratio of 6∶1 (alcohol∶oil) in the presence of different concentrations of mCLEAs (prepared using 0.1 mg protein/mg MNP–NH_2_, 100 mmol sulphate/mg protein, 200 mM glutaraldehyde, no detergent). After incubating the reaction mixtures containing mCLEAs (at 0.5, 1, 2 and 5% w/w of oil) for 24 h at 30°C, conversions of 64.0, 76.1, 81.5 and 82.2%, respectively, were obtained. These data indicate that a very low concentration of the biocatalyst (1% w/w of oil) is suitable to catalyze the transesterification of olive oil.

**Figure 6:**
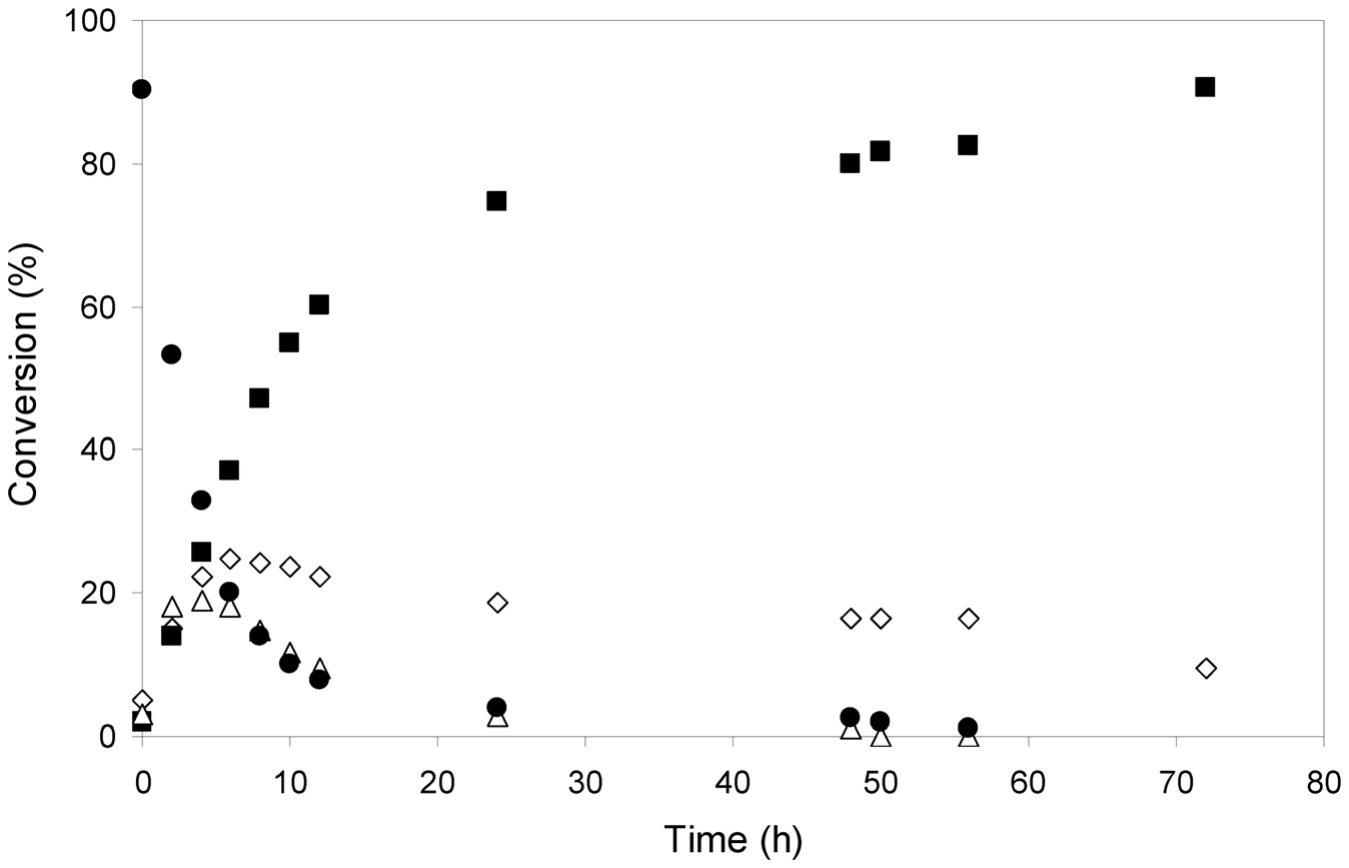
Time-course of biodiesel (FAPEs) conversion catalyzed by mCLEAs of CALB. The reaction mixture containing 200 mg olive oil, 2-propanol (6∶1 alcohol∶oil molar ratio) and 1% (w/w of oil) mCLEAs was incubated for 72 h at 30°C with rotational mixing. Samples (10 µl) were withdrawn at the indicated times and analyzed by HPLC. Triglycerides (•), FAPEs (▪), diglycerides (▵) and monoglycerides with free fatty acids (⋄).

**Figure 7:**
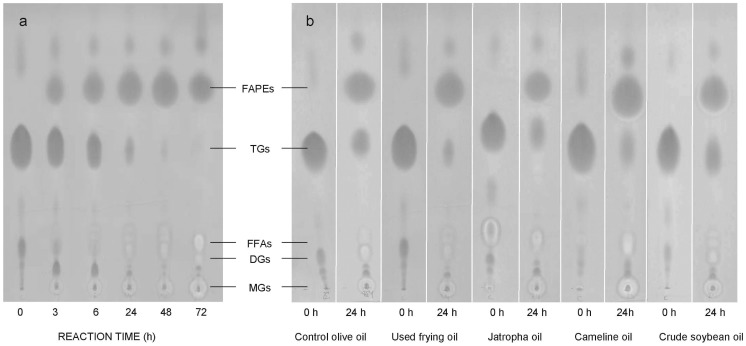
Analysis by TLC of biodiesel (FAPEs) conversion using mCLEAs of CALB. The reaction mixture and conditions were the same as in Fig. 6, except that (a) waste frying olive oil and (b) different vegetable oils were used as substrate. Olive oil was included as a control substrate. Triglycerides (TGs), diglycerides (DGs), monoglycerides (MGs) and free-fatty acids (FFAs).

The time-course of the reaction was assessed during 72 h for both control olive oil and waste frying oil. Samples were withdrawn at time intervals and analyzed by either HPLC (olive oil) or TLC (waste frying oil). HPLC data revealed the appearance of diglycerides, monoglycerides and free fatty acids (Fig. 6) as intermediates in the synthesis of biodiesel (fatty acid propyl esters, FAPEs). These intermediates could be also observed in samples analyzed by TLC and stained with Coomassie Blue (Fig. 7a), indicating that this versatile semi-quantitative method can be useful to determine the reaction progress with time by following changes in substrate and products concentrations. It is worth mentioning that TLC analysis followed by plate revealing with Coomassie Blue permits to analyze in parallel at least 10 samples in less than 1 h. In addition it allows the use of crude and/or recycled vegetable oils which could originate troubles when analyzed by HPLC.

The efficiency of low concentrations of mCLEAs to catalyze the conversion into biodiesel of other non-edible vegetable triglycerides was tested using jatropha, cameline and crude (unrefined) soybean oils. Samples of these crude oils were used without any pretreatment. The transesterification reaction with 2-propanol was carried out using 1% of biocatalyst (w/w oil) at 30°C for 72 h, as described previously for olive oils, and the resulting FAPEs were analyzed by TLC. As shown in Fig. 7b, similar bioconversions were observed in all cases after 24 h of transesterification reaction.

The immobilization of lipase to different carriers for biodiesel production has been studied previously with high conversion results (64–100%), although elevated concentrations of biocatalyst (5–15% w/w of oil) were used in most of the cases [49]–[52]. Even when lipase was immobilized on NH_2_-functionalized magnetic nanoparticles, requirements from 17.3 to 40% (w/w of oil) of biocatalyst were reported [18]–[20]. Only a few number of researchers described the use of lower concentrations of enzyme to catalyze the transesterification reaction, as Mendes et al. [53] who used 0.2% (w/w of oil) of enzyme, although they worked at 45°C with high alcohol∶oil molar ratios. It is well known that temperature not only increases kinetics but also can affect stability of the enzyme.

**Figure 8:**
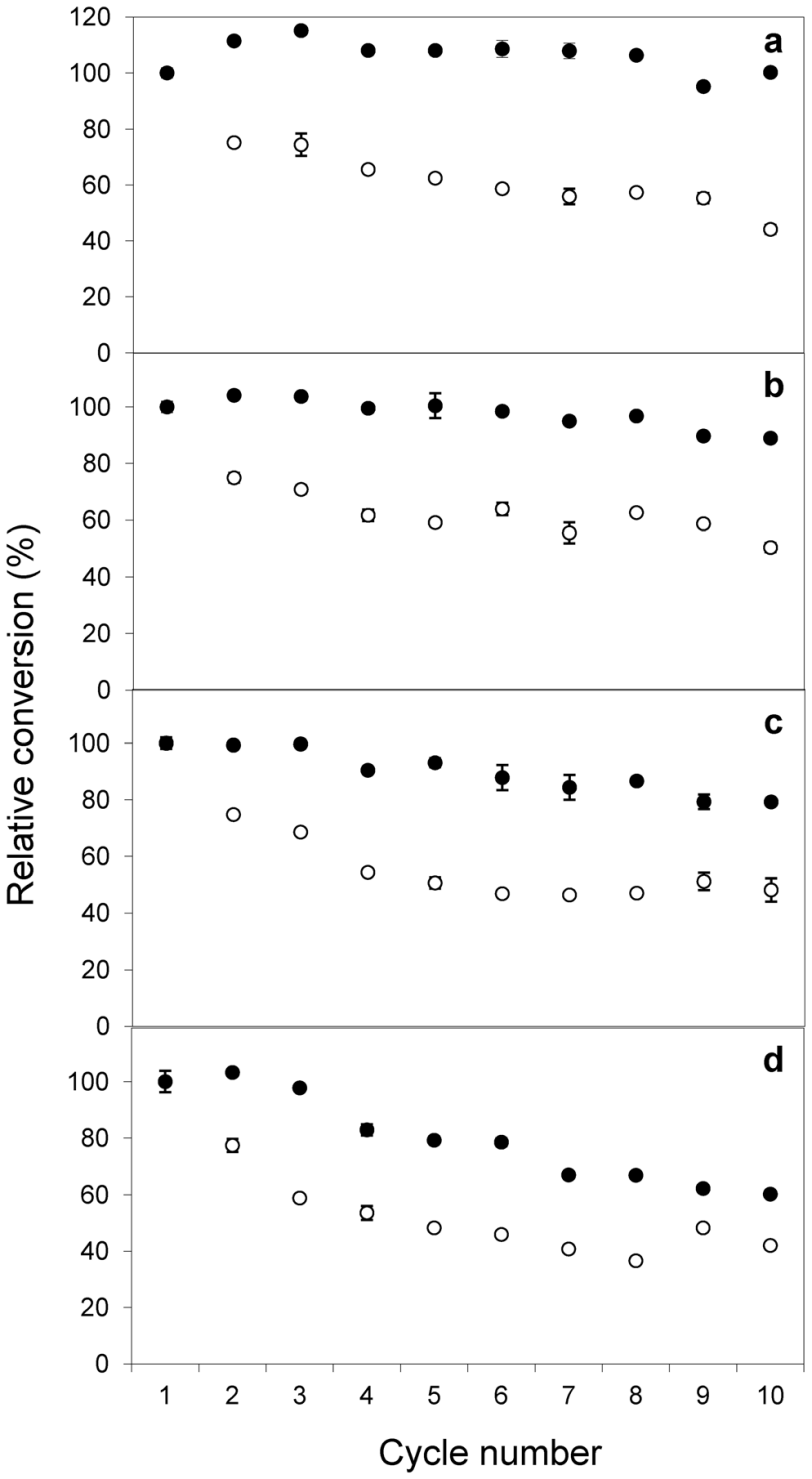
Reusability of mCLEAs of CALB at different temperatures. Biocatalyst was used in consecutive reaction cycles of 24 h for biodiesel (FAPEs) conversion from olive oil at: (a) 30°C, (b) 40°C, (c) 50°C and (d) 60°C. The reaction mixture and conditions were the same as in Fig. 6. Relative conversion is referred to the final conversion after the first cycle and was calculated using samples analyzed by HPLC. Two types of mCLEAs were tested which were prepared using 0.05 (◯) and 0.1 mg protein/mg MNP-NH_2_ (•).

The stability and reusability of mCLEAs of CALB was investigated by reusing the biocatalyst for several consecutive reaction cycles at temperatures from 30 to 60°C. After each cycle (24 h) the biocatalyst was recovered from the reaction mixture using a magnetic field. Two different catalysts, prepared using 0.05 and 0.1 mg CALB/mg MNP–NH_2_, were tested. The biocatalyst with lower concentration of lipase not only showed a 4-fold lower transesterification activity but it was also considerably less stable than its counterpart containing 0.1 mg protein/mg MNP–NH_2_ (Fig. 8). Using the latter, the 24 h-conversion was preserved for at least 10 consecutive reaction cycles without observing any significant loss of catalytic capacity. Although stability decreased with the increase in temperature, the highly-loaded biocatalyst maintained more than 60% of the activity after 10 cycles at 60°C.

Wang et al. [20] observed that the retained activity of their lipase catalyst decreased by more than 70% after 10 consecutive cycles of reaction at 40°C; Xie and Ma [18], [19] observed a decrease of 50% activity after the fifth cycle of catalysis using *Thermomyces lanuginosus* lipase immobilized on MNPs for the production of biodiesel at high temperatures (45–50°C). Our data reveal that the combination of cross-linked enzyme aggregates with MNPs results in a robust biocatalyst which is able to catalyze the synthesis of biodiesel at low enzyme concentration, and is more stable than other lipase preparations reported so far. The effectiveness of this biocatalyst for the synthesis of biosurfactants was also proved recently in a previous work from our group [54].

Novel enzyme engineering techniques are being applied for the improvement of catalytic efficiencies of CALB. Thus, Agarwal and coworkers [55] introduced a pothoactivatable molecule on the surface of the enzyme, which allowed the modulation of the enzyme conformation by light stimulation, obtaining an increase in 24% of CALB catalytic activity. A combination of protein engineering with mCLEAs formation could result in an easily recoverable and reusable enzyme with improved enzymatic activity and high stability, which could considerably increase the robustness and applicability of the biocatalyst.

## Conclusions

In this work we report an efficient, facile and economical method to prepare mCLEAs of *Candida rugosa* lipase B by cross-linking with glutaraldehyde CALB aggregates onto the surface of MNPs functionalized with -NH_2_ groups. The resulting magnetic biocatalyst shows an enhanced stability and maintains the superparamagnetic behavior typical of MNPs, which allows for easy separation and reusability in consecutive catalytic cycles without apparent loss of activity. The utility of this robust biocatalyst for the efficiently production of biodiesel has been demonstrated although it could be also used to obtain other highly valuable compounds, such as biosurfactants, flavoring and aromatic compounds or bioactive compounds.

## Supporting Information

S1 Fig Effect of detergents on enzymatic activity. (a) Relative esterase activity of soluble CALB; (b) Oil transesterification activity of mCLEAs: (•) 0.1 mg CALB/mg MNP−NH_2_; (◯) 0.4 mg CALB/mg MNP−NH_2_.
(TIF)

S2 Fig Analysis of unbound protein after the different immobilization steps: (a) MNP-CALB (50 µg CALB/mg MNP-NH_2_ in absence of precipitant agent); (b) mCLEAs (100 µg CALB/mg MNP-NH_2_). Cont: control of offered protein (CALB, molecular mass  = 33.5 kDa); UR: unretained protein after 2 h of cross-linking; 1: first wash with PBS; 2: second wash with PBS; 3: third wash with PBS; BH_4_: unretained protein after NaBH_4_ reduction; NaCl: unretained protein after washing with 2 M NaCl; TX: unretained protein after washing with 1% (v/v) Triton X-100; B: liquid phase after incubating the MNP-CALB complex for 5 min at 100°C.
(TIF)

S1 Video Synthesis of magnetic nanoparticles.
(MPG)

## References

[1] Bajaj A, Lohan P, Jha PN, Mehrotra R (2010) Biodiesel production through lipase catalyzed transesterification: An overview. J Mol Catal B Enzym 62:9–14.

[2] Leung DYC, Wu X, Leung MKH (2010) A review on biodiesel production using catalyzed transesterification. Appl Energy 87:1083–1095.

[3] Bisen PS, Sanodiya BS, Thakur GS, Baghel RK, Prasad GB (2010) Biodiesel production with special emphasis on lipase-catalyzed transesterification. Biotechnol Lett 32:1019–1030. 20401680 10.1007/s10529-010-0275-z

[4] Ren Y, Rivera JG, He L, Kulkarni H, Lee DK, et al (2011) Facile, high efficiency immobilization of lipase enzyme on magnetic iron oxide nanoparticles via a biomimetic coating. BMC Biotechnol 11:63. 21649934 10.1186/1472-6750-11-63 PMC3212977

[5] Idris A, Bukhari A (2012) Immobilized *Candida antarctica* lipase B: Hydration, stripping off and application in ring opening polyester synthesis. Biotechnol Adv 30:550–563. 22041165 10.1016/j.biotechadv.2011.10.002

[6] Netto CGCM, Andrade LH, Toma HE (2009) Enantioselective transesterification catalysis by *Candida antarctica* lipase immobilized on superparamagnetic nanoparticles. Tetrahedron: Asymmetry 20:2299–2304.

[7] Ngo TPN, Li A, Tiew KW, Li Z (2013) Efficient transformation of grease to biodiesel using highly active and easily recyclable magnetic nanobiocatalyst aggregates. Bioresour Technol 145:233–239. 23298767 10.1016/j.biortech.2012.12.053

[8] Netto CGCM, Toma HE, Andrade LH (2013) Superparamagnetic nanoparticles as versatile carriers and supporting materials for enzymes. J Mol Catal B: Enzym 85–86:71–92.

[9] Verma ML, Barrow CJ, Puri M (2013) Nanobiotechnology as a novel paradigm for enzyme immobilisation and stabilisation with potential applications in biodiesel production. Appl Microbiol Biotechnol 97:23–39. 23132346 10.1007/s00253-012-4535-9

[10] Prabhavathi Devi BLA, Guo Z, Xu X (2009) Characterization of cross-linked lipase aggregates. J Am Oil Chem Soc 86:637–642.

[11] Schoevaart R, Wolbers MW, Golubovic M, Ottens M, Kieboom APG, et al (2004) Preparation, optimization, and structures of cross-linked enzyme aggregates (CLEAs). Biotechnol Bioeng 87:754–762. 15329933 10.1002/bit.20184

[12] Contesini FJ, Figueira JdA, Kawaguti HY, Fernandes PCdB, Carvalho PdO, et al (2013) Potential applications of carbohydrases immobilization in the food industry. Int J Mol Sci 14:1335–1369. 23344046 10.3390/ijms14011335 PMC3565324

[13] Sheldon RA (2007) Cross-linked enzyme aggregates (CLEAs): stable and recyclable biocatalysts. Biochem Soc Trans 35:1583–1587. 18031271 10.1042/BST0351583

[14] Sheldon RA (2011) Characteristic features and biotechnological applications of cross-linked enzyme aggregates (CLEAs). Appl Microbiol Biotechnol 92:467–477. 21887507 10.1007/s00253-011-3554-2 PMC3189406

[15] Sheldon RA, van Pelt S (2013) Enzyme immobilization in biocatalysis: why, what and how. Chem Soc Rev 42:6223–6235. 23532151 10.1039/c3cs60075k

[16] Talekar S, Ghodake V, Ghotage T, Rathod P, Deshmukh P, et al (2012) Novel magnetic cross-linked enzyme aggregates (magnetic CLEAs) of alpha amylase. Bioresour Technol 123:542–547. 22944488 10.1016/j.biortech.2012.07.044

[17] Talekar S, Joshi A, Joshi G, Kamat P, Haripurkar R, et al (2013) Parameters in preparation and characterization of cross-linked enzyme aggregates (CLEAs). RSC Adv 3:12485–12511.

[18] Xie W, Ma N (2009) Immobilized lipase on Fe_3_O_4_ nanoparticles as biocatalyst for biodiesel production. Energy Fuels 23:1347–1353.

[19] Xie W, Ma N (2010) Enzymatic transesterification of soybean oil by using immobilized lipase on magnetic nano-particles. Biomass Bioenergy 34:890–896.

[20] Wang X, Dou P, Zhao P, Zhao C (2009) Immobilization of lipases onto magnetic Fe_3_O_4_ nanoparticles for application in biodiesel production. ChemSusChem 2:947–950. 19780103 10.1002/cssc.200900174

[21] Han JY, Kim HK (2011) Transesterification using the cross-linked enzyme aggregate of *Photobacterium lipolyticum* lipase M37. J Microbiol Biotechnol 21:1159–1165. 22127127 10.4014/jmb.1106.06048

[22] Lai J-Q, Hu ZL, Sheldon RA, Yang Z (2012) Catalytic performance of cross-linked enzyme aggregates of *Penicillium expansum* lipase and their use as catalyst for biodiesel production. Process Biochem 47:2058–2063.

[23] Cruz-Izquierdo Á, Picó EA, Anton-Helas Z, Boeriu CG, Llama MJ, et al (2012) Lipase immobilization to magnetic nanoparticles: methods, properties and applications for biobased products. New Biotechnol 29S:S100–S101.

[24] Tudorache M, Nae A, Coman S, Parvulescu V (2013) Strategy of cross-linked enzyme aggregates onto magnetic particles adapted to the green design of biocatalytic synthesis of glycerol carbonate. RSC Adv 3:4052–4058.

[25] Morales MdP, Veintemillas-Verdaguer S, Montero MI, Serna CJ, Roig A, et al (1999) Surface and internal spin canting in γ-Fe_2_O_3_ nanoparticles. Chem Mater 11:3058–3064.

[26] Shen X-C, Fang X-Z, Zhou Y-H, Liang H (2004) Synthesis and characterization of 3-aminopropyltriethoxysilane-modified superparamagnetic magnetite nanoparticles. Chem Lett 33:1468–1469.

[27] Liu ZL, Liu YJ, Yao KL, Ding ZH, Tao J, et al (2002) Synthesis and magnetic properties of Fe_3_O_4_ nanoparticles. J Mater Synth Process 10:83–87.

[28] del Campo A, Sena T, Lellouche J-P, Bruce IJ (2005) Multifunctional magnetite and silica–magnetite nanoparticles: synthesis, surface activation and applications in life sciences. J Magn Magn Mater 293:33–40.

[29] Mateo C, Fernández-Lorente G, Abian O, Fernández-Lafuente R, Guisán JM (2000) Multifunctional epoxy supports: a new tool to improve the covalent immobilization of proteins. The promotion of physical adsorptions of proteins on the supports before their covalent linkage. Biomacromolecules 1:739–745. 11710205 10.1021/bm000071q

[30] Laemmli UK (1970) Cleavage of structural proteins during the assembly of the head of bacteriophage T4. Nature 277:680–685. 10.1038/227680a0 5432063

[31] Gao L, Xu J-H, Li X-J, Liu Z-Z (2004) Optimization of *Serratia marcescens* lipase production for enantioselective hydrolysis of 3-phenylglycidic acid ester. J Ind Microbiol Biotechnol 31:525–530. 15549608 10.1007/s10295-004-0182-1

[32] Holčapek M, Jandera P, Fischer J, Prokeš B (1999) Analytical monitoring of the production of biodiesel by high-performance liquid chromatography with various detection methods. J Chromatogr A 858:13–31. 10544888 10.1016/s0021-9673(99)00790-6

[33] Samakawa T, Kaieda M, Matsumoto T, Ban K, Kondo A, et al (2000) Pretreatment of immobilized *Candida antarctica* lipase for biodiesel fuel production from plant oil. J Biosci Bioeng 90:180–183. 16232839

[34] Nakamura K, Handa S (1984) Coomassie brilliant blue staining of lipids on thin-layer plates. Anal Biochem 142:406–410. 6084960 10.1016/0003-2697(84)90484-6

[35] Harris LA, Goff JD, Carmichael AY, Riffle JS, Harburn JJ, et al (2003) Magnetite nanoparticle dispersions stabilized with triblock copolymers. Chem Mater 15:1367–1377.

[36] Sahoo Y, Goodarzi A, Swihart MT, Ohulchanskyy TY, Kaur N, et al (2005) Aqueous ferrofluid of magnetite nanoparticles: Fluorescence labeling and magnetophoretic control. J Phys Chem 109:3879–3885. 10.1021/jp045402y 16851439

[37] Lee J, Na HB, Kim BC, Lee JH, Lee B, et al (2009) Magnetically-separable and highly-stable enzyme system based on crosslinked enzyme aggregates shipped in magnetite-coated mesoporous silica. J Mater Chem 19:7864–7870.

[38] Ramírez LP, Landfester K (2003) Magnetic polystyrene nanoparticles with a high magnetite content obtained by miniemulsion processes. Macromol Chem Phys 204:22–31.

[39] Maity D, Agrawal DC (2007) Synthesis of iron oxide nanoparticles under oxidizing environment and their stabilization in aqueous and non-aqueous media. J Magn Magn Mater 308:46–55.

[40] Bayramoğlu G, Kiralp S, Yilmaz M, Toppare L, Arica MY (2008) Covalent immobilization of chloroperoxidase onto magnetic beads: Catalytic properties and stability. Biochem Eng J 38:180–188.

[41] Yong Y, Bai YX, Li YF, Lin L, Cui YJ, et al (2008) Preparation and application of polymer-grafted magnetic nanoparticles for lipase immobilization. J Magn Magn Mater 320:2350–2355.

[42] Huang SH, Liao MH, Chen DH (2003) Direct binding and characterization of lipase onto magnetic nanoparticles. Biotechnol Progr 19:1095–1100. 10.1021/bp025587v 12790688

[43] Sohn OJ, Kim CK, Rhee JI (2008) Immobilization of glucose oxidase and lactate dehydrogenase onto magnetic nanoparticles for bioprocess monitoring system. Biotechnol Bioprocess Eng 13:716–723.

[44] Gupta P, Dutt K, Misra S, Raghuwanshi S, Saxena RK (2009) Characterization of cross-linked immobilized lipase from thermophilic mould *Thermomyces lanuginosus* using glutaraldehyde. Bioresour Technol 100:4074–4076. 19403306 10.1016/j.biortech.2009.03.076

[45] Fernández-Lorente G, Palomo JM, Mateo C, Munilla R, Ortiz C, et al (2006) Glutaraldehyde cross-linking of lipases adsorbed on aminated supports in the presence of detergents leads to improved performance. Biomacromolecules 7:2610–2615. 16961324 10.1021/bm060408+

[46] Fernández-Lorente G, Palomo JM, Cabrera Z, Fernández-Lafuente R, Guisán JM (2007) Improved catalytic properties of immobilized lipases by the presence of very low concentrations of detergents in the reaction medium. Biotechnol Bioeng 97:242–250. 17054124 10.1002/bit.21230

[47] Liu Y-Y, Xu J-H, Hu Y (2000) Enhancing effect of Tween-80 on lipase performance in enantioselective hydrolysis of ketoprofen ester. J Mol Catal B Enzym 10:523–529.

[48] López-Serrano P, Cao L, van Rantwijk F, Sheldon RA (2002) Cross-linked enzyme aggregates with enhanced activity: application to lipases. Biotechnol Lett 24:1379–1383.

[49] Dizge N, Keskinler B (2008) Enzymatic production of biodiesel from canola oil using immobilized lipase. Biomass Bioenergy 32:1274–1278.

[50] Azócar L, Ciudad G, Heipieper HJ, Muñoz R, Navia R (2010) Improving fatty acid methyl ester production yield in a lipase-catalyzed process using waste frying oils as feedstock. J Biosci Bioeng 109:609–614. 20471601 10.1016/j.jbiosc.2009.12.001

[51] Charpe TW, Rathod VK (2011) Biodiesel production using waste frying oil. Waste Manage 31:85–90. 10.1016/j.wasman.2010.09.003 20889327

[52] Iso M, Chen B, Eguchi M, Kudo T, Shrestha S (2001) Production of biodiesel fuel from triglycerides and alcohol using immobilized lipase. J Mol Catal B Enzym 16:53–58.

[53] Mendes AA, Giordano RC, Giordano RdLC, Castro HFd (2011) Immobilization and stabilization of microbial lipases by multipoint covalent attachment on aldehyde-resin affinity: Application of the biocatalysts in biodiesel synthesis. J Mol Catal B: Enzym 68:109–115.

[54] López C, Cruz-Izquierdo A, Picó EA, García-Bárcena T, Villarroel N, et al (2014) Magnetic biocatalysts and their uses to obtain biodiesel and biosurfactants. Front Chem 2:72. 25207271 10.3389/fchem.2014.00072 PMC4144358

[55] Agarwal PK, Schultz C, Kalivretenos A, Ghosh B, Broedel SE (2012) Engineering a hyper-catalytic enzyme by photoactivated conformation modulation. J Phys Chem Lett 3:1142–1146.

