# Supplementary figures and images for: Magnetic Cross-Linked Enzyme Aggregates (mCLEAs) of Candida antarctica Lipase: An Efficient and Stable Biocatalyst for Biodiesel Synthesis
Source: PLoS One. 2014 Dec 31;9(12):e115202. doi: 10.1371/journal.pone.0115202 (PMC4281201; doi:10.1371/journal.pone.0115202)

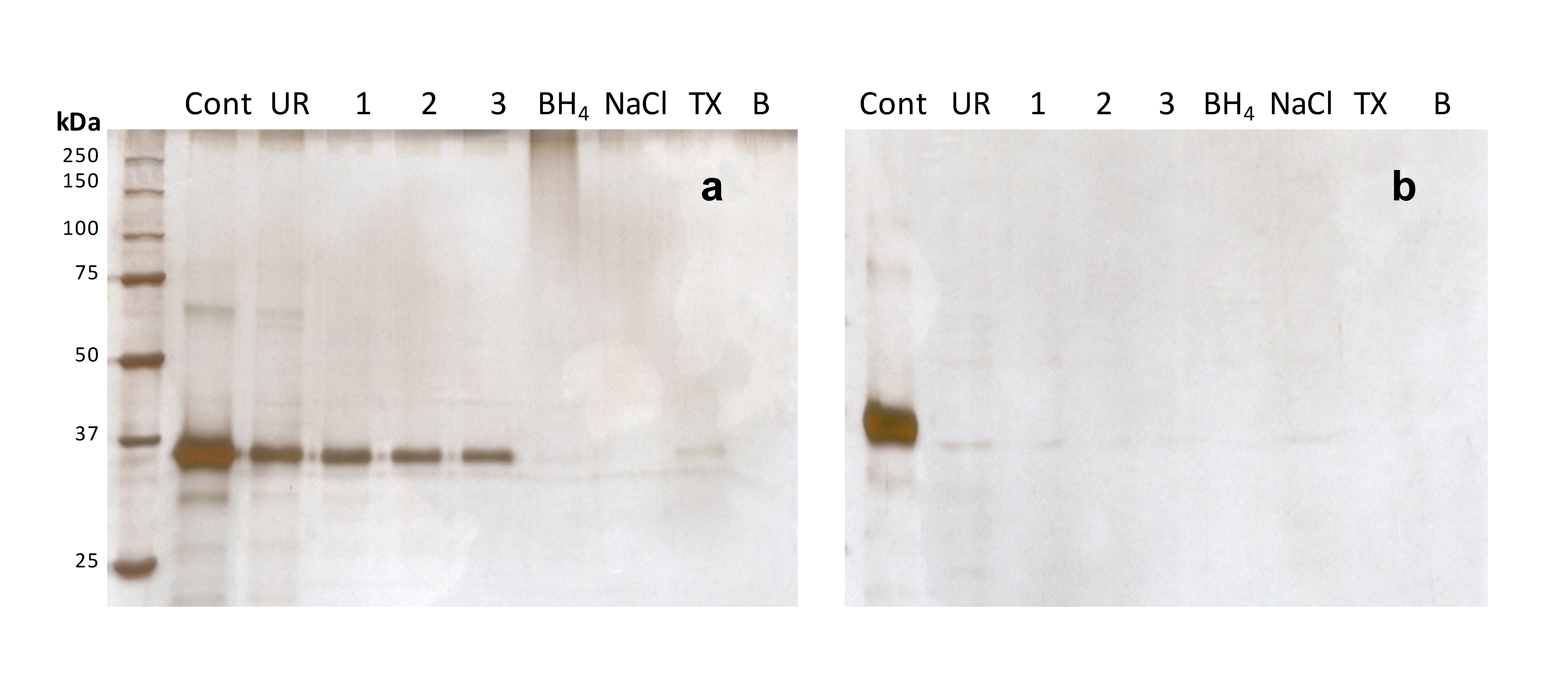

Supplement: S2 Fig — Analysis of unbound protein after the different immobilization steps: (a) MNP-CALB (50 µg CALB/mg MNP-NH2 in absence of precipitant agent); (b) mCLEAs (100 µg CALB/mg MNP-NH2). Cont: control of offered protein (CALB, molecular mass = 33.5 kDa); UR: unretained protein after 2 h of cross-linking; 1: first wash with PBS; 2: second wash with PBS; 3: third wash with PBS; BH4: unretained protein after NaBH4 reduction; NaCl: unretained protein after washing with 2 M NaCl; TX: unretained protein after washing with 1% (v/v) Triton X-100; B: liquid phase after incubating the MNP-CALB complex for 5 min at 100°C. (TIF) [file pone.0115202.s002.tif]
